# Diagnosis of arboviruses using indirect sandwich IgG ELISA in horses from the Brazilian Amazon

**DOI:** 10.1186/1678-9199-20-29

**Published:** 2014-07-03

**Authors:** Alexandre do Rosário Casseb, Márcio Roberto Teixeira Nunes, Sueli Guerreiro Rodrigues, Elizabeth Salbé Travassos da Rosa, Livia Medeiros Neves Casseb, Samir Manssor Moraes Casseb, Sandro Patroca da Silva, Érika Dayane Leal Rodrigues, Pedro Fernando da Costa Vasconcelos

**Affiliations:** 1 Institute of Health and Livestock Production, Federal Rural University of the Amazon (UFRA), Av. Presidente Tancredo Neves, 2501, Belém, Pará State 66077-901, Brazil; 2 Department of Arbovirology and Hemorrhagic Fevers, Evandro Chagas Institute, Ananindeua, Pará State, Brazil; 3 Institute of Biological Sciences, Federal University of Pará State, Belém, Pará, Brazil; 4 Department of Pathology, Pará State University, Belém, Pará State, Brazil

**Keywords:** Horses, Arboviruses, ELISA

## Abstract

**Background:**

The Amazon as a whole is the largest reservoir of arboviruses worldwide, while the Brazilian Amazon hosts the largest variety of arboviruses isolated to date. In this study, the results of an indirect sandwich IgG ELISA, standardized for 19 arbovirustypes circulating among horses in Brazilian Amazon, were compared to results of the hemagglutination inhibition test. A screening test assessed the conditional probability distribution and a Pearson linear correlation test determined the correlation strength among the absorbance values recorded for viruses from the same family.

**Findings:**

Sensitivity varied between 40.85 and 100%; the specificity was low and ranged from 39.71 to 67.0%; and the accuracy varied between 41 and 65.2%. The test developed in this study yielded a large number of serological cross-reactions.

**Conclusions:**

The test can be employed to detect IgG antibodies within one arbovirus family; however, the hemagglutination test or other more specific techniques, such as the serum neutralization test in mice or the plaque-reduction neutralization test, are essential complementary methods for positive cases.

## Findings

The expression ‘arthropod-borne virus’ was introduced in 1942 to describe a group of viruses that were propagated by arthropods and biologically transmitted to vertebrate hosts. Two decades later, the International Committee on the Nomenclature of Viruses officially recommended the term ‘arbovirus’ to denominate the viruses maintained through cycles involving hematophagous arthropod vectors and vertebrate hosts [1].

Considered as a whole, the Amazon region is the largest reservoir of arboviruses worldwide. To date, 196 out of at least 200 arbovirus types found throughout Brazil were identified in the Brazilian Amazon, where the largest variety have been isolated, while many of the viral strains have not been found in any other location [2-4].

For more than two decades, the serological study of arboviruses has been restricted to such classical techniques as hemagglutination inhibition (HI), complement fixation (CF) and serum neutralization in mice (SN). Among these, the HI test in microplates is recommended as a routine serological test [2].

**Figure 1:**
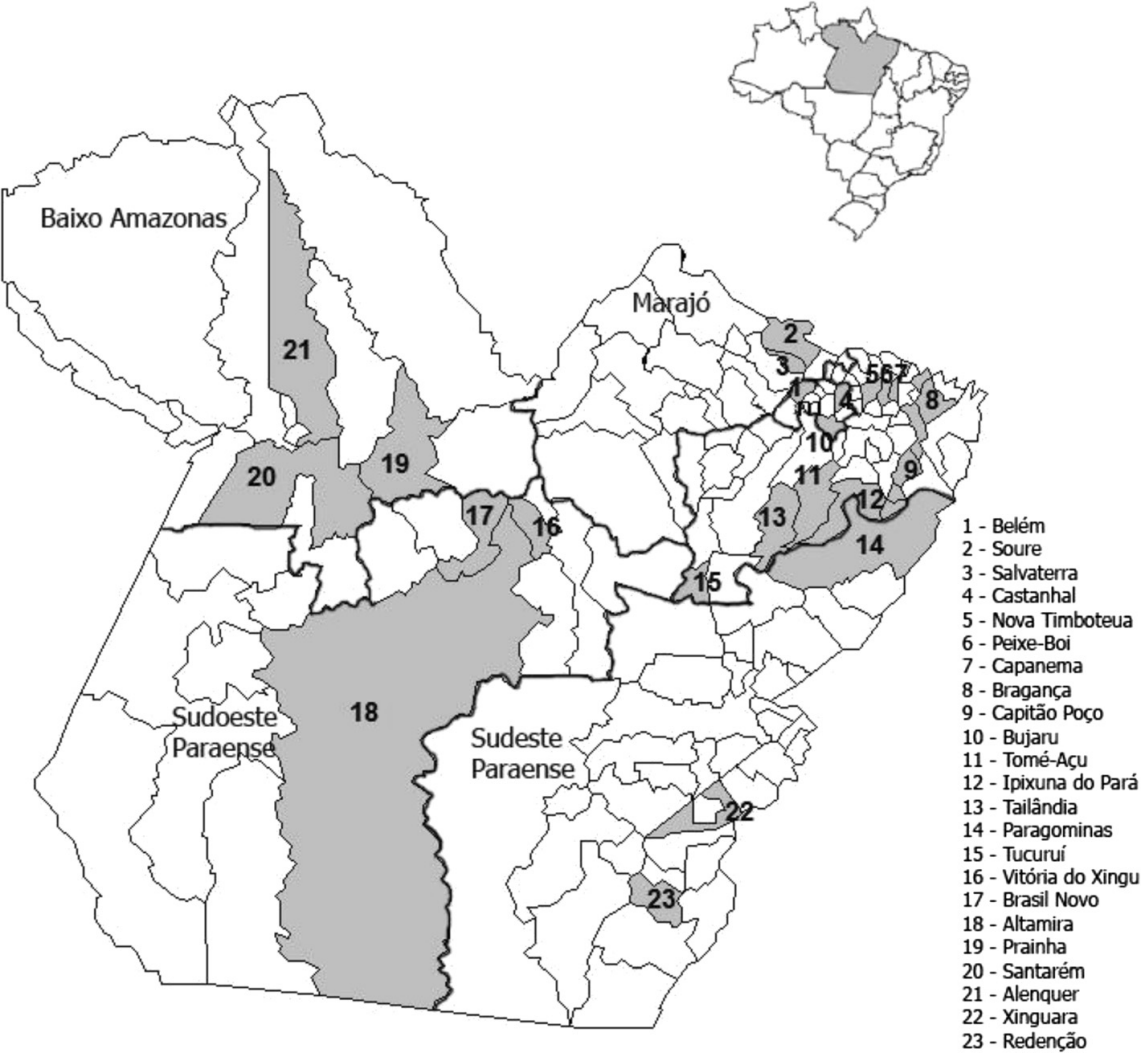
Map of the State of Pará depicting the mesoregions (thick line) and municipalities (solid gray area) where serum from horses were collected.

The enzyme-linked immunosorbent assay (ELISA) method is widely used for the serological diagnosis of arboviruses because it is not only very sensitive but also easy to perform and standardize; thus, it is able to strengthen the power of some of the classical techniques. The sensitivity and practicality of the ELISA systems are also evident in tests for detecting immunoglobulin G (IgG), which allow many serum samples to be assayed in a single day because the sample amounts can be small and do not require any pretreatment. These facts provided the basis of this study, in which an indirect sandwich IgG ELISA method was standardized to diagnose infections by 19 arboviruses types circulating among horses in the Brazilian Amazon.Blood was collected from the animals throughout 2009 in the state of Pará, Brazil (Figure 1). The animals subjected to bleeding were aged two years old or older, had been born and raised at the sample collection site and had not been vaccinated against arboviruses.

The ELISA technique was standardized for each of the arbovirus types by adapting the protocols described for the dengue virus (DENV) [5]. The test was performed with antigens of 19 different arbovirus types isolated in Brazil (Table 1) from the collection of the Arbovirology and Hemorrhagic Fevers Section of the Evandro Chagas Institute, Ananindeua, Pará, Brazil. The positive-control serum samples exhibited titers ≥ 20 in the HI test, and preferentially presented monotypic reactions [6,7]. The negative-control samples exhibited negative results in the HI test (with titers < 20 for all of the 19 viruses under study). The conjugate used was the peroxidase-conjugated affinity pure rabbit anti-equine IgG (Jackson Immunoresearch Laboratories, USA).

**Table 1:** Arbovirus antigens comprising 19 arbovirus types isolated in Brazil and belonging to the collection of the arbovirology and hemorrhagic fevers section of the Evandro Chagas Institute, Brazil

**Family**	**Genus**	**Viral antigens**
*Togaviridae*	*Alphavirus*	*Eastern equine encephalitis virus* (EEEV)
		*Western equine encephalitis virus* (WEEV)
		*Mayaro virus* (MAYV)
		*Mucambo virus* (MUCV)
*Flaviviridae*	*Flavivirus*	*Yellow fever virus* (YFV)
		*Ilheus virus* (ILHV)
		*Saint Louis encephalitis virus* (SLEV)
		*Cacipacore virus* (CPCV)
		*Bussuquara virus* (BSQV)
		*Rocio virus* (ROCV)
*Bunyaviridae*	*Orthobunyavirus*	*Guaroa virus* (GROV)
		*Maguari virus* (MAGV)
		*Tacaiuma virus* (TCMV)
		*Utinga virus* (UTIV)
		*Belem virus* (BLMV)
		*Caraparu virus* (CARV)
		*Oropouche virus* (OROV)
		*Catu virus* (CATUV)
	*Phlebovirus*	*Icoaraci virus* (ICOV)

After the standardization of the technique, a total of 232 serum samples of the horses for each of the 19 investigated arbovirus types were used. The results of the indirect sandwich ELISA for the IgG detection were compared to the HI test through a conditional probability distribution, using a screening test to establish the sensitivity, specificity and accuracy of each of the tests. A Pearson linear correlation test was used to verify the correlation strength among the absorbance values obtained using the enzyme immunoassay, that is, the possibility of cross-reactions among the antibodies to viruses belonging to the same family; the accepted correlation values (r) varied from -1 ≤ r ≤ 1, as calculated using the software BioEstat 5.0 [8].

The optimal serum dilution, which varied according to the virus family employed, was 1:100 for the family *Togaviridae* and 1:200 for the families *Bunyaviridae* and *Flaviviridae.* The conjugated antibodies were used at a dilution of 1:10,000.

The sensitivity varied between 40.85 (ILHV) and 100% (ICOV and BLMV); the specificity was low and ranged from 39.71 (ROCV) to 67.0% (MAYV); and the accuracy varied between 41 (ILHV) and 65.2% (MAYV).

The Pearson correlation coefficient(r) varied for the *Flaviviridae* family from 0.78 between CPCV and VSLE to 0.95 between CPCV and BSQV; for the *Togaviridae* family, r varied from 0.89 between EEEV and MUCV to 0.96 between EEEV and WEEV. In the *Bunyaviridae* family, r varied from 0.71 between GROV and UTIV to 0.96 between MAGV and TCMV.

An investigation of anti-arbovirus IgG antibodies has already been performed using ELISA in humans and domestic animals [9-11]. In this study, the serum dilution varied as a function of the arbovirus family and proved to be crucial for standardizing the indirect sandwich IgG ELISA method. An additional crucial factor for standardizing this technique was the dilution of the antibody-enzyme conjugate and the antigen.

When defining an ELISA test cutoff, the most important feature is to select serum samples from animals that are actually infected and from those that have never come into contact with the investigated virus [12]. Although the present study took this requirement into account, the degree of cross-reactivity among the investigated arbovirus species was high. To increase the test sensitivity, antigen purification and/or the use of highly specific antibodies may be needed; however, the production of stock and purified viral antigens for ELISA using classical methods is expensive and time-consuming, especially when a viral agent does not reach high multiplication titers in cell cultures [13].

We stress that the HI test detects both IgM and IgG and does not distinguish between them; thus, it is possible that some of the positive results of the HI test were not matched by the indirect sandwich IgG ELISA test used in this study, thus decreasing the calculated sensitivity of ELISA. Another important factor is that all of the investigated animals were aged more than two years, which implies a higher probability for the animals to have contacted a larger number and wider diversity of arboviruses, thus increasing the odds of cross-reactions [14].

The interpretation of serological tests for arboviruses must be performed cautiously because the tests might exhibit cross-reactions among the antigenically most-related arbovirus types in the investigated families, especially in horses with multiple exposures to arthropods and, thus, with a greater risk of contamination by several arboviruses [15].

The indirect sandwich ELISA test developed in this study for 19 arbovirus types in horses exhibited a large number of serological cross-reactions. Therefore, we conclude that the protocol developed herein can be used to detect IgG within the same arbovirus family, but the method cannot distinguish among the arbovirus species belonging to a given family. Thus, indirect sandwich IgG ELISA must be used together with the HI test or other more specific techniques, such as the SN test or the plaque reduction neutralization test (PRNT).

## Ethics committee approval

All of the procedures, which involved newborn (2–3 days old) Swiss albino mice and domesticated animals, were performed with utmost strictness to avoid any unnecessary suffering. The present study was submitted to and approved by the Ethics Committee on Animal Research (CEPAN) of the Evandro Chagas Institute (IEC; ruling 054/2009 CEPAN/IEC).

## Competing interests

The authors declare that there are no competing interests.

## Authors’ contributions

ARC took part in sample collection, serological tests and manuscript writing. LMNC carried out serological tests and statistical analysis. SPS contributed in sample collection and serological tests. SMMC, MRTN, SGR and ESTR performed serological tests. ÉDLR performed serological tests and wrote the article. PFCV participated in writing and reviewing the article. All authors read and approved the final manuscript.
